# Remarkably Enhanced Room-Temperature Hydrogen Sensing of SnO_2_ Nanoflowers via Vacuum Annealing Treatment

**DOI:** 10.3390/s18040949

**Published:** 2018-03-23

**Authors:** Gao Liu, Zhao Wang, Zihui Chen, Shulin Yang, Xingxing Fu, Rui Huang, Xiaokang Li, Juan Xiong, Yongming Hu, Haoshuang Gu

**Affiliations:** 1 Hubei Collaborative Innovation Center for Advanced Organic Chemical Materials—Hubei Key Laboratory of Ferro & Piezoelectric Materials and Devices, Faculty of Physics & Electronic Sciences, Hubei University, Wuhan 430062, China; Liugao@stu.hubu.edu.cn (G.L.); chenzihui@stu.hubu.edu.cn (Z.C.); yangsl@hgnu.edu.cn (S.Y.); fuxingxing@stu.hubu.edu.cn (X.F.); Huangrui@stu.hubu.edu.cn (R.H.); lixk@stu.hubu.edu.cn (X.L.); xiongjuana@163.com (J.X.); huym@hubu.edu.cn (Y.H.); 2 School of Electronic Information, Huanggang Normal University, Huanggang 438000, China

**Keywords:** hydrogen sensing, SnO_2_, annealing, nanoflowers, hydrothermal

## Abstract

In this work, SnO_2_ nanoflowers synthesized by a hydrothermal method were employed as hydrogen sensing materials. The as-synthesized SnO_2_ nanoflowers consisted of cuboid-like SnO_2_ nanorods with tetragonal structures. A great increase in the relative content of surface-adsorbed oxygen was observed after the vacuum annealing treatment, and this increase could have been due to the increase in surface oxygen vacancies serving as preferential adsorption sites for oxygen species. Annealing treatment resulted in an 8% increase in the specific surface area of the samples. Moreover, the conductivity of the sensors decreased after the annealing treatment, which should be attributed to the increase in electron scattering around the defects and the compensated donor behavior of the oxygen vacancies due to the surface oxygen adsorption. The hydrogen sensors of the annealed samples, compared to those of the unannealed samples, exhibited a much higher sensitivity and faster response rate. The sensor response factor and response rate increased from 27.1% to 80.2% and 0.34%/s to 1.15%/s, respectively. This remarkable enhancement in sensing performance induced by the annealing treatment could be attributed to the larger specific surface areas and higher amount of surface-adsorbed oxygen, which provides a greater reaction space for hydrogen. Moreover, the sensors with annealed SnO_2_ nanoflowers also exhibited high selectivity towards hydrogen against CH_4_, CO, and ethanol.

## 1. Introduction

Hydrogen (H_2_) is regarded as one of the most promising clean energies owing to its environmentally friendly combustion properties, its high combustion temperature, its low minimum ignition energy, its wide inflammable range, etc. The development of hydrogen energy-related technologies have become significant examples of new energy technology [1]. However, hydrogen is dangerous owing to its small molecular size. It easily leaks out from containers and pipelines during production, storage, transportation, and application processes. Once hydrogen concentrations increase to ~4–75% in air, fires or explosions and thus catastrophic accidents can occur [2,3]. As a result, the detection of hydrogen leakages and the monitoring of hydrogen concentrations in confined indoor environments are crucial for the security assurance in hydrogen-related fields.

Hydrogen is colorless, odorless, and tasteless, so humans cannot perceive it. Therefore, fast and sensitive hydrogen gas sensors are required to timely detect hydrogen leakage incidents. Among the developed hydrogen sensors, those based on metal oxide semiconductors have attracted tremendous attention due to their high sensitivity, long lifetime, low cost, and good compatibility with silicon microfabrication [4,5]. The sensing mechanism of such devices can be attributed to the surface redox reaction between adsorbed oxygen species and target H_2 _ molecules, leading to the release of trapped electrons around the adsorbed oxygen and an increase in the conductivity of those materials [6,7]. However, traditional semiconductor-based hydrogen sensors usually require a high working temperature of 150–400 °C to maintain their high sensitivity, which may decrease the stability and lifetime of the sensors due to the heat-induced growth of metal oxide grains and unrecoverable chemical reduction of the sensing layers by hydrogen. Furthermore, high-temperature operations also risk ignition when highly inflammable H_2_ is detected and the power consumption of the sensor systems increases, which limits the application of these sensors in wireless sensor networks.

Recently, low-dimensional semiconductor oxide nanomaterials, such as thin films, nanowires, and nanorods, have been widely investigated as high-performance hydrogen sensing materials [8,9,10,11,12]. The much higher specific surface area and the low-dimensional nanoscale electron transportation pathway makes them highly sensitive to surface reactions between adsorbed oxygen species and target hydrogen gases. Therefore, such nanomaterials exhibit a much better sensing performance than traditional bulk materials, including higher sensitivity, a lower limit of detection (LOD), a faster response behavior, and most importantly, a much lower operating temperature.

SnO_2_ is an *n*-type semiconductor material with good chemical stability, a wide band gap, and low-cost materials, which have exhibited great advantages in terms of building stable and sensitive gas sensor systems [13,14,15]. For example, SnO_2_ thin films synthesized via sol–gel have exhibited fast and highly sensitive hydrogen sensing at relatively low temperatures (100 °C) compared to ZnO, TiO_2_, CuO, and WO_3_ thin films [16]. Sputter-deposited Pt/SnO_2_ thin films demonstrated by Shahabuddin and co-workers also exhibited sensitive hydrogen sensing, but at 110 °C [17]. K. Choi et al. reported an on-chip hydrogen sensor based on SnO_x_ films on comb-shaped interdigital electrodes (IDEs) via reactive ion-assisted deposition, which showed a fast response to hydrogen gas at 150 °C [18]. Moreover, sensors based on SnO_2_ nanowires and nanorods have exhibited superior hydrogen sensing at relatively low temperatures because of their higher specific surface area compared to thin-film-based devices [19,20]. However, room-temperature hydrogen sensing of both SnO_2_ thin films and nanowire networks are still unsatisfactory due to the response time of up to several minutes. Although surface decoration with noble metal nanomaterials, such as Pd nanoparticles, and hydrogen splitting catalysis can enhance the room-temperature sensing performance of SnO_2_ nanowires, this method will substantially increase the fabrication cost of the sensors [14,15,21,22]. As a result, a low-cost and efficient method to improve the hydrogen response of SnO_2_ one-dimensional nanomaterials at room temperature is needed.

As reported, the hydrogen sensing performance of semiconductor oxide nanomaterials are closely related to surface defects such as oxygen vacancies, which are preferential adsorption sites for oxygen species [23]. Therefore, controlling the defect concentration and the adsorption of oxygen may be an efficient way of enhancing the hydrogen sensing of SnO_2_ nanomaterials. In this work, a simple, low-cost, and high-yield hydrothermal method was employed for synthesizing SnO_2_ nanoflowers consisting of co-cored single-crystal nanorods. The nanoflowers were integrated into nanorod membranes to fabricate hydrogen sensors. High-temperature annealing in vacuum conditions was applied to an as-fabricated device. The room-temperature hydrogen sensing of the devices was greatly enhanced after the vacuum annealing process. The sensing mechanism is discussed in detail here.

## 2. Materials and Methods

SnCl_4_·5H_2_O and absolute ethanol were purchased from Sinopharm chemical Reagent Co., Ltd. (Shanghai, China). All reagents were analytical grade and used as received without further purification. SnO_2_ nanoflowers were synthesized by the hydrothermal method. Firstly, 1.33 g SnCl_4_·5H_2_O solids was dissolved in 20 mL of an NaOH solution (1.26 M). The mixture was then continuously stirred for 10 min at room temperature. After that, 20 mL of absolute ethanol was added into the reaction system and further stirred to obtain a white translucent suspended solution. Five minutes later, the white translucent suspended solution was then transferred into a stainless-steel autoclave (60 mL). The system was then heated in an electric oven at 200 °C for 72 h and cooled naturally to room temperature. The product was washed several times with DI water and then collected after vacuum suction filtration. The as-prepared products were dried at 70 °C for 12 h.

The composition of the materials was examined with an X-ray diffractometer (XRD, Bruker D8A25, CuKα, λ = 1.5406 Å). The morphology and microstructure were characterized with a field-emission scanning electron microscope (FESEM, JEOL JSM7100F). X-ray photoelectron spectroscopy (XPS) experiments were conducted with a VG ESCALAB-MK electron spectrometer using Cu Kα radiation. Surface area and porosity were extracted with Brunauer–Emmett–Teller (BET) measurements using Quantachrome Nova 1200 with N_2_ as the adsorbate at liquid nitrogen temperature.

For the gas sensing test, ethanol and the as-obtained product, whose mass ratio was 10:1, were mixed. The suspension was spin-coated on the SiO_2_ quartz glass substrates and then dried at 80 °C. After that, the samples were annealed for 2 h at 300 °C in a vacuum condition, respectively. The Pt/Ti interdigital electrodes (IDEs) with a finger spacing of 100 μm and a thickness of 120 nm were then deposited on the top surface of the sensing layers by the DC magnetron sputtering method. After wire leading by copper wires, the sensors based on the SnO_2_ nanoflowers were obtained.

The room temperature gas sensing properties were measured by monitoring the change of the sensor resistance with a Keithley 2400 digital source meter at a working voltage of 5.0 V when the sensor was exposed to air or the test gas in homemade gas sensor testing systems. By this process, hydrogen gas with certain concentrations in air was quantitatively prepared. Hydrogen sensing behavior was determined by measuring the changes in electrical resistance and repeatedly altering the hydrogen-containing atmosphere and dry air.

## 3. Results and Discussions

### 3.1. Materials Characterizations

**Figure 1:**
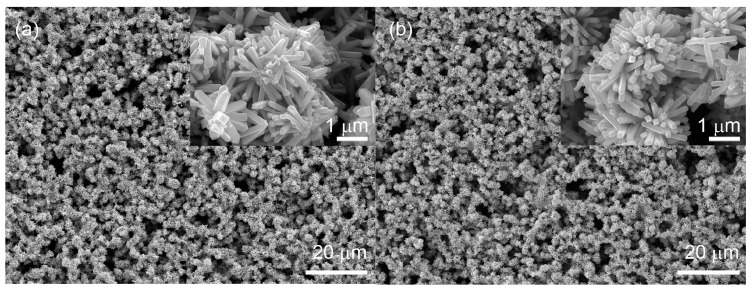
SEM images of as-synthesized SnO_2_ nanoflowers (**a**) before annealing and (**b**) annealed in a vacuum condition.

Figure 1 shows the SEM images of the as-synthesized products before and after heat treatments, which were spin-coated on the SiO_2_ quartz glass substrates. As shown in Figure 1a, the products synthesized by the hydrothermal method consisted of nanoflowers with several cuboid-like nanorods grown from the central core. The size of the nanorods was ~1.5 μm and 350 nm along the axial and radial directions, respectively. The diameter of the nanoflowers was approximately 2–3 μm. Due to the flower-like hierarchical microstructure of the particles, the membrane formed by the spin-coating method exhibited obviously porous structures, which are favorable for gas sensing applications because they provide high amounts of gas-adsorption sites and unobstructed gas diffusion pathways for both gas adsorption and desorption processes. Moreover, there was no obvious change in either the morphology of the membrane or the size of the nanoflowers after heat treatment in the vacuum condition (Figure 1b), indicating no further growth of grains after annealing treatment.

**Figure 2:**
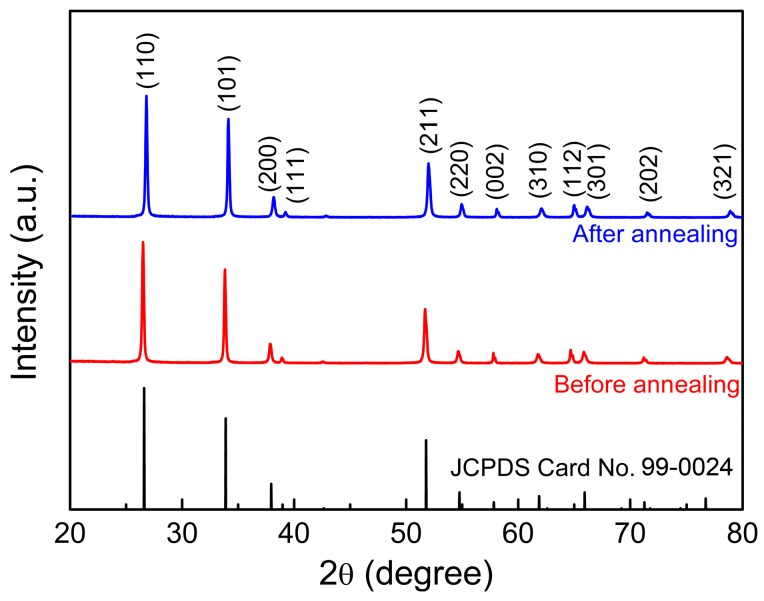
XRD patterns of as-synthesized SnO_2_ nanoflowers before and after annealing treatments.

The XRD patterns of the as-synthesized samples are shown in Figure 2. The diffraction peaks in all curves can be indexed to the tetragonal phase of the SnO_2_ structure according to the JCPDS Card No. 99-0024. The sharp and intense diffraction peaks indicate satisfactory crystallinity in the as-synthesized SnO_2_ nanoflowers. Moreover, the diffraction peaks of the annealed samples shifted towards higher degrees. The lattice parameters could be calculated according to the diffraction peaks as a = 0.4750 nm and c = 0.3190 nm for the untreated samples and a = 0.4740 nm and c = 0.3184 nm for the annealed samples, respectively. A slight shrink in unit cell volume (~0.6%) was induced by the annealing treatment. Such lattice contraction can be attributed to the increase in crystallinity and the release of strain, which might be induced during hydrothermal growth processes.

**Figure 3:**
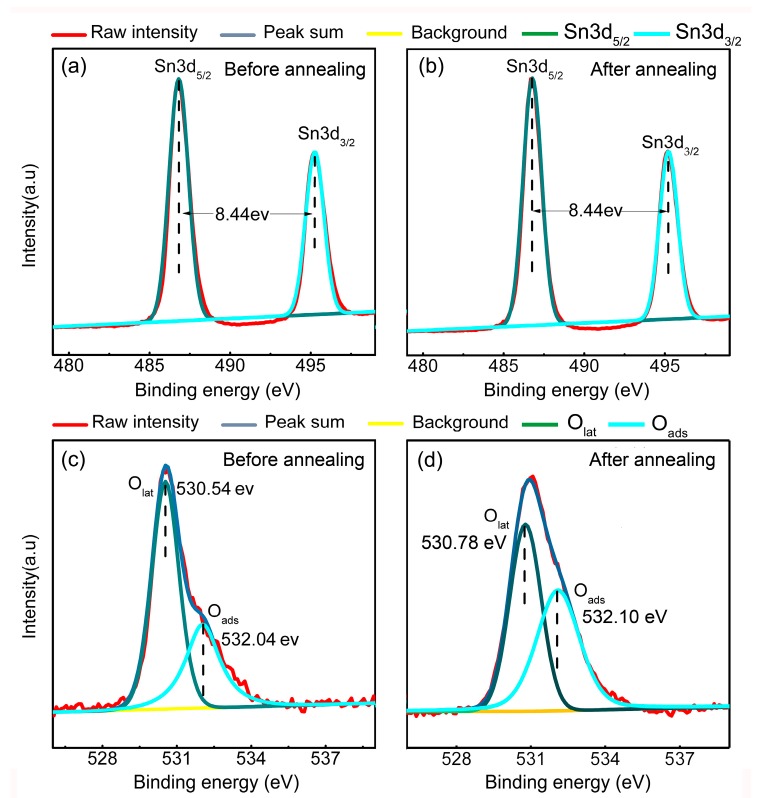
The XPS patterns of the SnO_2_ nanoflowers (**a**,**c**) before annealing and (**b**,**d**) after annealing.

Figure 3 shows the XPS analysis results of the O 1s and Sn 3d spectra for both samples. The obtained spectra surveys were divided into Gaussian components with a Shirley background with the software XPSPEAK. The Sn 3d spectrum is shown in Figure 3a,b, where two strong peaks can be found at ~495.22 eV for Sn 3d_3/2_ and 486.78 eV for 3d_5/2_. These results confirm that no Sn^2+^ was formed after the vacuum annealing treatments. Moreover, as shown in Figure 3c, the O 1s peaks exhibited two distinct peaks with binding energies around 530.54 and 532.04 eV, which could be attributed to the lattice oxygen (O_lat_) and adsorbed oxygen O_x_ (O_2ads_, O_ads_, or O_2ads_) ions, respectively [24]. After peak fitting, the ratio between the lattice oxygen and adsorbed oxygen could be estimated as ~65:35 according to the integral area of each peak. For samples annealed annealing in a vacuum condition at 300 °C, the raw data (red curve) of the O 1s peak shown in Figure 3d exhibits obvious broadening profile, indicating an increase in peak intensity for the adsorbed oxygen. After peak fitting, the ratio between the lattice oxygen and the adsorbed oxygen was ~59:41, which is much higher than that of the unannealed samples. As reported in our previous work, the oxygen vacancies are active sites due to the deficiency of lattice oxygen, which leads to a dangling bond in the surface area. The binding energy of the dissociative oxygen atoms on the oxygen vacancies were much lower than the other sites (lattice oxygen and cations) [23]. Thus, oxygen vacancies always serve as preferential adsorption sites. As vacuum annealing treatment will generate oxygen vacancies in the metal oxide nanomaterials, the remarkable increase in adsorbed oxygen after vacuum annealing treatment should be attributed to the increase in oxygen vacancies [25].

**Figure 4:**
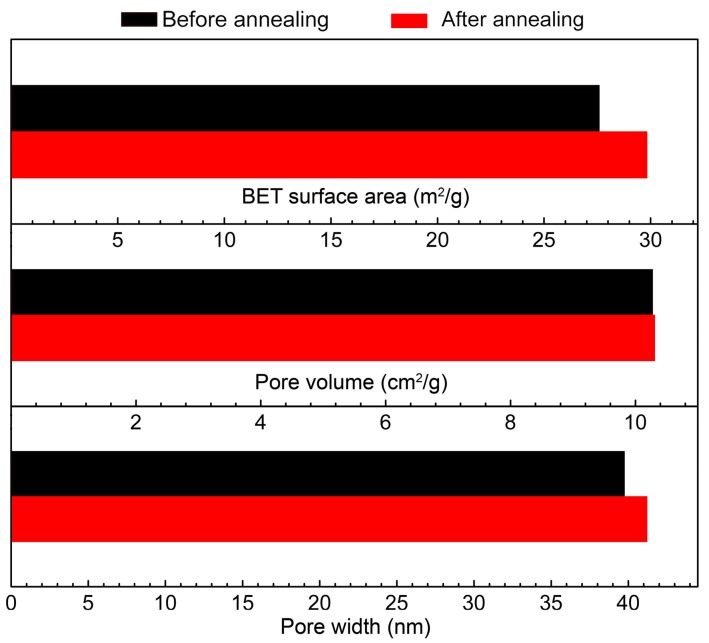
BET surface area, pore volume, and pore width of the as-synthesized SnO_2_ nanoflowers before and after annealing treatment.

Figure 4 shows the BET characterization results of the as-synthesized SnO_2_ nanoflowers before and after the vacuum annealing treatment. The specific surface area of the samples shows a slight increase from ~27.6 to 29.8 m^2^/g after the vacuum annealing treatment. Meanwhile, the pore volume and width of the samples were also increased after the annealing treatment. Such increases in the specific surface area and pore width may improve the gas adsorption/desorption behavior and the diffusion in the sensing layers. This variation in the BET results may be attributed to the slight shrink of the SnO_2_ nanoflowers that occurred after the annealing treatment, which is also suggested by the XRD results, and may have led to the increase in pore width due to the decreased volume density of the SnO_2_ nanoflowers in the sensing layers.

### 3.2. Sensor Performance

**Figure 5:**
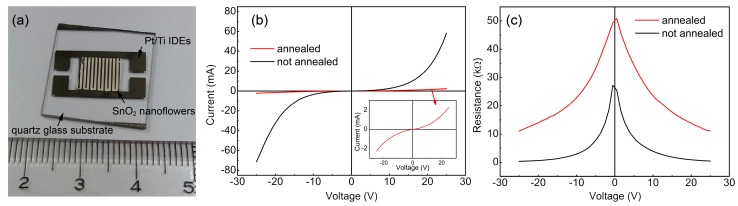
(**a**) The photo image, (**b**) the I-V characteristics, and (**c**) the R-V curves of the as-fabricated hydrogen sensors based on the SnO_2_ nanoflowers before and after annealing treatment.

Figure 5a shows a schematic diagram and optical image of an as-fabricated sensors based on SnO_2_ nanoflowers. The I-V characteristics of the devices based on unannealed and annealed SnO_2_ nanoflowers were measured in air and in a hydrogen-containing atmosphere, respectively. As shown in Figure 5b, the I-V curve of the unannealed device exhibited typical Schottky-type behavior, indicating that electron transportation was dominated by the thermionic emission of the electrons at the interfaces between adjacent SnO_2_ nanorods. The inset picture shows the magnified view of the I-V curve of the annealed sample, of which the nonlinear Schottky-type electron transportation behavior was reduced. Considering that the metal-semiconductor contact should be an ohmic contact due to the similar work function of Ti (4.33 eV) and SnO_2_ (4.5 eV), the variation in conducting behavior can likely be attributed to the improvement of the interface contact between the adjacent SnO_2_ nanorods after annealing treatment, which decreased the interface energy barriers [26]. Moreover, the current of the devices with the annealed SnO_2_ nanoflowers was much lower than that of the unannealed ones at the same voltage level. Figure 5c shows the variation in resistance with the applied voltage of both sensors. As shown, the resistance of the device with the annealed SnO_2_ nanoflowers, compared with that of the unannealed samples, exhibited a much higher value. As reported, the oxygen vacancies in the *n*-type semiconductor oxides will lead to an increase in electron density due to their donor behavior [23]. Meanwhile, such defects may also result in electron scattering and thus decrease the carrier mobility of the semiconductor materials. Considering that the donor behavior of the oxygen vacancies could be compensated by the surface-adsorbed oxygen species, which always serve as the electron trapping centers, the decrease in conductivity is likely mainly due to the enhanced electron scattering in the semiconductors.

**Figure 6:**
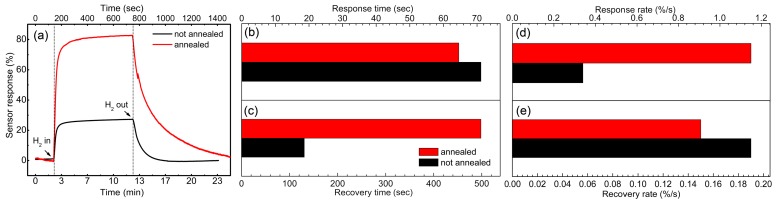
The hydrogen response of as-fabricated sensors based on the SnO_2_ nanoflowers before and after annealing treatments. (**a**) Time-dependent variation of resistance with hydrogen in and out; (**b**,**c**) Response and recovery time; (**d**,**e**) Response and recovery rate.

To understand the dynamic hydrogen response of both devices, the time-dependent variation of resistance was measured with an applied voltage of 5 V, when the atmosphere was switched from one containing air to one containing hydrogen. Due to the difference in the initial resistance, the sensor response factor (*S*) was defined for direction comparison of the sensing performance of both devices, which can be interpreted as

$$S=\frac{R_{air}-R_{hydrogen}}{R_{air}}\times 100\%$$

where *R_air_* and *R_hydrogen_* represents the steady-state resistance value of the devices in air- and hydrogen-containing atmospheres, respectively. Figure 6a shows the room-temperature resistance response to 1000 ppm of hydrogen for both sensors within one cycle of a response and recovery process. As shown, the annealed samples, compared to the unannealed samples, exhibited a much more sensitive room-temperature response to hydrogen gas. The maximum sensor response was ~80.2% and 27.1%, respectively. Moreover, the response time (*t_res_*) and recovery time (*t_rec_*) (time used for achieve 90% of total response/recovery) of both sensors were calculated and shown in Figure 6b,c. Both sensors exhibited fast room-temperature hydrogen response with a response time of ~62 and 71 s for the annealed and unannealed samples, respectively. However, annealing treatment led to a great increase in the recovery time of the sensors, which is ~500 s and 130 s, respectively. Because the sensor response is different between these two samples, the comparison of average response rate and recovery rate could be more appropriate for understanding the change induced by the annealing treatment to the sensing behavior of SnO_2_ nanoflowers. As shown in Figure 6d, the average response rate (calculated as *v_res_* = 0.9*S_max_*/*t_res_*) of the annealed samples was much higher than that of the unannealed ones (1.15 and 0.34%/s, respectively). By contrast, the average recovery rate (*V_rec_* = 0.9*S_max_*/*t_rec_*) exhibited a slight decrease from ~0.19 to 0.15%/s after annealing treatment, as shown in Figure 6e.

As reported in the literature, the room-temperature hydrogen-sensing behavior of SnO_2_ and other semiconductor oxide nanomaterials can likely be attributed to the reaction between the surface-adsorbed oxygen species and the incoming hydrogen [3,4,6,7]. The reaction process could be interpreted as

$$\begin{array}{l} O_{2}(g)\to 2O(\mathrm{ads})\\ O(\mathrm{ads})+e^{-}\to O^{-}(\mathrm{ads})\\ H_{2}+O^{-}(\mathrm{ads})\to H_{2}O(g)+e^{-} \end{array}.$$

Firstly, the oxygen gas can become adsorbed on the surface of the SnO_2_ (usually at the site of the oxygen vacancies), and this can then lead to the formation of electron trapping centers for capturing the electrons from the conduction bands. As a result, the charge carrier depletion layer will be formed at the surface area of the nanorods. Due to the one-dimensional nanostructure of the sensing materials, the surface depletion layer may lead to an increase in the bulk resistance of the nanorods. Therefore, the sensing layer will be at a high-resistance state under an ambient condition. Once the sensing layers are exposed to hydrogen gas, the adsorbed oxygen species will react with the hydrogen molecular and release the trapped electrons to the conduction band. As a result, the charge carrier density may return to a relatively higher level, resulting in the elimination of the depletion layer. As a result, the resistance will return to a lower level. Because the density of released electron is closely related to the reacted hydrogen molecular, the variation in resistance can be used to reflect the variation in hydrogen concentration in the ambient environment. 

In this work, the SnO_2_ nanoflowers after vacuum annealing treatment contained a high amount of adsorbed oxygen according to the XPS results, which provided many active sites for the hydrogen sensing reaction. Therefore, the sensitivity of the annealed samples was much higher than that of the unannealed samples. The much higher concentration of an active site can also increase the reaction rate during the response process, such that the response rate of the annealed samples is much faster. However, the recovery process was less affected by annealing treatment, which could be attributed to the similar re-adsorption process for both annealed and unannealed samples. Moreover, the higher specific surface area, pore volume, and pore width may also contribute to the enhancement in the hydrogen sensing performance of the annealed samples. 

**Figure 7:**
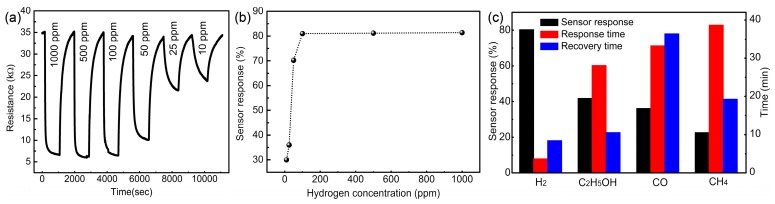
The room-temperature hydrogen response of the device with annealed SnO_2_ nanoflowers. (**a**) The time-dependent variation of sensor resistance toward different concentration of hydrogen in air; (**b**) The relationship between the sensor response and hydrogen concentration; (**c**) Response and recovery times, of the sensor toward 1000 ppm H_2_, C_2_H_5_OH, CO, and CH_4_.

To comprehensively estimate the room-temperature sensing performance of the sensors with annealed SnO_2_ nanoflowers, the sensor response behavior toward various concentration of hydrogen in air were measured. As shown in Figure 7a, the gas sensors displayed reversible and reproducible real-time responses. The sensor response increased as the hydrogen concentration increased below 100 ppm, while the response remained unchanged when the hydrogen concentration was higher than 100 ppm (Figure 7b). These results suggest that the response of the sensor reached the saturated state for hydrogen concentration higher than 100 ppm. However, the response time of the sensors toward a lower concentration of hydrogen was much longer, which was likely due to the slow reaction process of lower concentrations. Figure 7c shows the selective response of the hydrogen sensor against three common interference gases—CO, CH_4_, and ethanol. All measurements were taken with a gas concentration of 1000 ppm. As shown, the sensors exhibited a greater response to hydrogen than to the other three interference gases. The response and recovery times of the sensors towards hydrogen were also much shorter than they were towards the other gases. These results confirm the highly selective hydrogen gas sensing performance of these SnO_2_-nanoflower-based devices.

## 4. Conclusions

SnO_2_ nanoflowers consisting of co-cored SnO_2_ nanorods with tetragonal lattice structures were synthesized by a hydrothermal method. The relative content of surface-adsorbed oxygen species increased after vacuum annealing treatment, which could be attributed to the increased concentration of surface oxygen vacancies and thus the increased preferential adsorption sites for oxygen in ambient environment. The specific surface areas were slightly increased by ~8% after heat treatment. The nanoflowers were employed as room-temperature hydrogen sensing materials used to build a semiconductor-type hydrogen sensor by coating the SnO_2_ nanoflowers onto a quartz glass substrate, followed by the deposition of Pt/Ti IDEs. The conductivity of the sensors decreased after annealing treatment, which could be attributed to the increase in carrier scattering around the defects. Moreover, the sensors with annealed samples, compared to those of the unannealed samples, exhibited a much higher hydrogen sensitivity and a faster response rate. The sensor response factor and response rate increased from 27.1% to 80.2% and 0.34%/s to 1.15%/s, respectively. This remarkable enhancement in sensing performance could be attributed to the higher amount of surface-adsorbed oxygen and larger specific surface areas induced by the annealing treatment. Moreover, the sensors with annealed SnO_2_ nanoflowers exhibited high selectivity towards hydrogen against CH_4_, CO, and ethanol.
